# Supplementary material for: Geometry of visuospatial working memory information in miniature gaze patterns
Source: Nat Hum Behav. 2023 Dec 18;8(2):336–48. doi: 10.1038/s41562-023-01737-z (PMC10896725; doi:10.1038/s41562-023-01737-z)
Supplement: Supplementary file 1 — Reporting Summary [file 41562_2023_1737_MOESM1_ESM.pdf]

nature portfolio

Corresponding author(s): Juan Linde-Domingo and Bernhard Spitzer

Last updated by author(s): Aug 30, 2023

Reporting Summary

Nature Portfolio wishes to improve the reproducibility of the work that we publish. This form provides structure for consistency and transparency in reporting. For further information on Nature Portfolio policies, see our Editorial Policies and the Editorial Policy Checklist.

Statistics

For all statistical analyses, confirm that the following items are present in the figure legend, table legend, main text, or Methods section.

n/a	Confirmed	
☐	☒	The exact sample size (n) for each experimental group/condition, given as a discrete number and unit of measurement
☐	☒	A statement on whether measurements were taken from distinct samples or whether the same sample was measured repeatedly
☐	☒	The statistical test(s) used AND whether they are one- or two-sided<br>*Only common tests should be described solely by name; describe more complex techniques in the Methods section.*
☒	☐	A description of all covariates tested
☐	☒	A description of any assumptions or corrections, such as tests of normality and adjustment for multiple comparisons
☐	☒	A full description of the statistical parameters including central tendency (e.g. means) or other basic estimates (e.g. regression coefficient) AND variation (e.g. standard deviation) or associated estimates of uncertainty (e.g. confidence intervals)
☐	☒	For null hypothesis testing, the test statistic (e.g. F, t, r) with confidence intervals, effect sizes, degrees of freedom and P value noted<br>*Give P values as exact values whenever suitable.*
☒	☐	For Bayesian analysis, information on the choice of priors and Markov chain Monte Carlo settings
☒	☐	For hierarchical and complex designs, identification of the appropriate level for tests and full reporting of outcomes
☐	☒	Estimates of effect sizes (e.g. Cohen's d, Pearson's r), indicating how they were calculated

Our web collection on statistics for biologists contains articles on many of the points above.

Software and code

Policy information about availability of computer code

Data collection	The experiment was run using Psychophysics Toolbox Version 3 (PTB; Brainard & Vision, 1997) and its incorporated Eyelink Toolbox version for PTB Version 3 (Cornelissen et al., 2002) in MATLAB 2017a (MathWorks).
Data analysis	All analyses were run using MATLAB 2018a (MathWorks).

For manuscripts utilizing custom algorithms or software that are central to the research but not yet described in published literature, software must be made available to editors and reviewers. We strongly encourage code deposition in a community repository (e.g. GitHub). See the Nature Portfolio guidelines for submitting code & software for further information.

Data

Policy information about availability of data

All manuscripts must include a data availability statement. This statement should provide the following information, where applicable:

- Accession codes, unique identifiers, or web links for publicly available datasets
- A description of any restrictions on data availability
- For clinical datasets or third party data, please ensure that the statement adheres to our policy

The data that support this study are available at https://gin.g-node.org/lindedomingo/mpib_memoreye

Research involving human participants, their data, or biological material

Policy information about studies with human participants or human data. See also policy information about sex, gender (identity/presentation), and sexual orientation and race, ethnicity and racism.

Reporting on sex and gender	Fifty-five participants (31 female, 24 male) took part in the experiment.
Reporting on race, ethnicity, or other socially relevant groupings	N/A -- no such data were collected (besides age and sex), and they played no role in participant recruitment
Population characteristics	Participants (young adults of any gender) were recruited from the general population of the city of Berlin (Germany) and surrounding areas with an interest in participating in scientific studies, in a age range of 18-35 years (mean age 26.95 ± 3.98 years).
Recruitment	We recruited young adult participants of any sex or gender. Potential participants were informed about receiving a compensation of €10 per hour plus a bonus based on task performance (€5 bonus if four out of five randomly selected memory reports were correct). Written informed consent was obtained from all participants prior to participation.
Ethics oversight	Deutsche Gesellschaft für Psychologie (DGPs), Bonn, Germany

Note that full information on the approval of the study protocol must also be provided in the manuscript.

Field-specific reporting

Please select the one below that is the best fit for your research. If you are not sure, read the appropriate sections before making your selection.

☒ Life sciences ☐ Behavioural & social sciences ☐ Ecological, evolutionary & environmental sciences

For a reference copy of the document with all sections, see nature.com/documents/nr-reporting-summary-flat.pdf

Life sciences study design

All studies must disclose on these points even when the disclosure is negative.

Sample size	Pilot experiments in our lab with similar stimulus materials showed that stimulus orientation was robustly reflected in gaze position in a sample of n = 20 participants during a simple WM maintenance period. Since the present experiment additionally included a second maintenance period that occurred only in half of the trials, we approximately doubled the sample size for the present study (n = 55 participants were recruited, of which n = 41 remained for analysis, see below). While no formal power analysis was conducted, post-hoc Bayesian Anlayses confirmed that the sample size was sufficient both to detect evidence for the presence and for the absence of an effect (e.g., Fig. 3)
Data exclusions	Two participants (both wearing glasses) were excluded due to difficulties in acquiring a stable eye-tracking signal, and one participant was excluded because they reported feeling unwell during the experimental session. Of the remaining participants, we excluded n = 9 for failing to perform above chance level in each of the two memory tests ($p < 0.05$, Binomial test against 50% correct responses). Finally, after preprocessing the eye-tracking data, we excluded n = 2 participants for whom more than 15% of the data had to be rejected due to blinks and other recording artifacts. After this, n = 41 participants remained for analysis.
Replication	N/A - The study includes no direct replication attempt of a previous finding. Aspects of our results that conceptually align with earlier findings are described as such in the manuscript text.
Randomization	The experiment was a within-subjects design without distinct experimental groups. The within-subjects task conditions were randomized as stated in Methods
Blinding	N/A -- The experiment was a within-subjects design where each participant performed the same variants of a behavioral task (blinding not applicable)

Reporting for specific materials, systems and methods

We require information from authors about some types of materials, experimental systems and methods used in many studies. Here, indicate whether each material, system or method listed is relevant to your study. If you are not sure if a list item applies to your research, read the appropriate section before selecting a response.

Materials & experimental systems

n/a	Involved in the study
☒	☐ Antibodies
☒	☐ Eukaryotic cell lines
☒	☐ Palaeontology and archaeology
☒	☐ Animals and other organisms
☒	☐ Clinical data
☒	☐ Dual use research of concern
☒	☐ Plants

Methods

n/a	Involved in the study
☒	☐ ChIP-seq
☒	☐ Flow cytometry
☒	☐ MRI-based neuroimaging
